# Juvenile fibromyalgia, a frequently missed disorder: a case report and literature review

**DOI:** 10.1192/j.eurpsy.2023.1633

**Published:** 2023-07-19

**Authors:** T. P. V. Alves, I. Cardoso, R. Amorim, I. Carrilho, T. Correia, I. Aguiar, Z. Correia

**Affiliations:** 1 Department of Psychiatry, Centro Hospitalar do Médio Tejo, Tomar; 2 Liaison Child Psychiatry Team; Pediatric Chronic Pain Multidisciplinary Consultation; Department of Child Psychiatry and Mental Health of Children and Adolescents; 3 Pediatric Chronic Pain Multidisciplinary Consultation; Department of Phisical Medicine and Rehabilitation; 4 Pediatric Chronic Pain Multidisciplinary Consultation; Department of Pediatrics; 5 Liaison Child Psychiatry Team; Department of Child Psychiatry and Mental Health of Children and Adolescents; 6 Liaison Child Psychiatry Team; Director of the Department of Child Psychiatry and Mental Health of Children and Adolescents, Centro Hospitalar Universitário do Porto - Centro Materno-Infantil do Norte (CHUP-CMIN), Porto, Portugal

## Abstract

**Introduction:**

The clinical features of juvenile fibromyalgia were first described by Yunus & Masi in 1985. In the US, it is estimated that about 6% of adolescents between 15 and 19 years of age suffer from juvenile fibromyalgia. However, this entity remains “a poorly defined disorder”, being excluded from the main diagnostic classification systems.

**Objectives:**

The goal of our work is to present and discuss a case-based review of juvenile fibromyalgia.

**Methods:**

We present a case of chronic pain in pediatric age, referred to a multidisciplinary chronic pain consultation. Through the analysis of this case, we review the concept of juvenile fibromyalgia and its pathophysiology, the risk factors, the diagnostic criteria, the recent evidence for the treatment of these cases and the prognosis of this disorder.

**Results:**

We describe the case of an 11-year-old female, who presented with widespread musculoskeletal pain, headaches, and sleep disturbances for a period over 3 months. At physical examination no significant alterations were found except for pain at palpation of the referred pain locations and at palpation of 11 typical fibromyalgia tender points. Complementary diagnostic exams were normal. The patient was referred to a multidisciplinary chronic pain consultation and was prescribed pharmacological therapy with antidepressants and a gabapentinoid and non-pharmacological therapy with a plan of physical exercises and psychotherapy.

**Conclusions:**

This case report demonstrates the importance of considering juvenile fibromyalgia in the differential diagnosis of pain in pediatric age, showing also the complexity involved in the assessment and treatment of these cases. This case also highlights the importance of multidisciplinary collaboration in the management of chronic pain.

**Disclosure of Interest:**

None Declared

